# Lactate acidosis and cardiac output during initial therapeutic cooling in asphyxiated newborn infants

**DOI:** 10.1371/journal.pone.0213537

**Published:** 2019-03-14

**Authors:** Vibeke Ramsgaard Eriksen, Simon Trautner, Gitte Holst Hahn, Gorm Greisen

**Affiliations:** Department of Neonatology, Copenhagen University Hospital–Rigshospitalet, Copenhagen, Denmark; Hopital Robert Debre, FRANCE

## Abstract

**Aim:**

We hypothesized that compromised cardiac output in asphyxiated infants may influence on the rate of disappearance of lactate due to insufficient perfusion.

**Methods:**

The study was a prospective, observational study, where infants with perinatal asphyxia who met the criteria for therapeutic hypothermia were included. Cardiac output, stroke volume and heart rate were measured by electrical velocimetry in 15 newborn infants with perinatal asphyxia during the first six hours of active therapeutic hypothermia. Results from routine blood samples were collected retrospectively. Cardiac parameters were also measured in 10 healthy, term infants after caesarian section. Cardiac parameters were compared between the asphyxiated group and the control group prior to and during hypothermia. Rate of disappearance of lactate was correlated to cardiac output in the asphyxiated infants.

**Results:**

Cardiac output was stable in the healthy infants from 0.5 to 6 hours postnatally. The infants with perinatal asphyxia had lower cardiac output prior to and during therapeutic hypothermia compared to the control group. Rate of disappearance of lactate was not related to cardiac output.

**Conclusion:**

An association between disappearance of lactate acidosis and low cardiac output was not confirmed. A low rate of disappearance of lactate may rather be an indicator of organ injury due to asphyxia.

## Introduction

Perinatal asphyxia may lead to multi-organ dysfunction [1–4], and a compromised cardiac output has been demonstrated in asphyxiated newborns [5,6]. As a neuroprotective therapy, therapeutic hypothermia has been implemented as standard practice. Cardiac output may be further compromised by therapeutic hypothermia [7–10].

Lactate acidosis is commonly observed after perinatal asphyxia. In critically ill newborn infants, prolonged lactate acidosis is associated with higher mortality [11,12], and in infants with hypoxic-ischemic encephalopathy the time for lactate to normalize has been shown to correlate with EEG grade and seizure burden [13]. Also, it has been suggested that lactate level may serve as a surrogate marker for suboptimal hemodynamic status of asphyxiated newborns treated with hypothermia [14].

In this observational study, we aimed to (i) describe asphyxia-induced compromised cardiac output and the effect of initiation of therapeutic hypothermia in asphyxiated newborns; and (ii) correlate the rate of disappearance of lactate with cardiac output in the early phase of therapeutic hypothermia. We hypothesized that compromised cardiac output in asphyxiated newborns may influence on the rate of disappearance of lactate due to insufficient perfusion.

## Patients and methods

### Patients

All fifteen infants, who were treated with therapeutic hypothermia after perinatal asphyxia between January and July 2016, were included, since patients were enrolled with deferred consent and all parents agreed to the study and use of data when asked at a late time. According to the local clinical guideline, the criteria for offering therapeutic hypothermia were ‘term’ birth (gestational age>36 weeks), acidosis in the first hour of life (pH<7.0 or base excess <-16 in arterial blood) and/or low 5 minute Apgar score (<6) and/or needed mechanical ventilation for more than 10 minutes after birth, and moderate or severe hypoxic-ischemic encephalopathy.

Treatment followed local clinal guidelines. In case of hypotension and signs of circulatory insufficiency (e.g. long capillary refill time, oliguria or persistent lactate acidosis) that could not be explained by hypovolemia the infants were treated with dopamine (2-15microg/kg/min) as first line therapy. Second line therapy included increased dopamine infusion rate or addition of norepinephrine.

A control group, consisting of ten term infants delivered by elective caesarian section (for convenience), was included. Indications for caesarian section were breech position or maternal request to ensure that the child was expected to be normal at birth. Postnatal exclusion criteria were congenital malformations, signs of asphyxia or need for ventilation.

Informed consent was obtained from all parents. In the control group 33 couples were informed about the study the day before the caesarian section, 15 declined to participate, and eight infants were excluded due to the fact, that we could only monitor one infant at a time. In this way 10 infants were included in the control group. The consent was obtained the day before the caesarian section and confirmed on the operative day. In the case group, the use of deferred consent was accepted by the research ethics committee, but data was used only if consent was obtained at a later time.

The study was approved by the Regional Ethical Committee (H-15010965).

### Ethical considerations

The study was approved by the Regional Ethical Committee, Region Hovedstaden, Denmark (H-15010965). In the control group the written consent was obtained the day before the caesarian section and confirmed on the operative day. In the case group the use of deferred consent was accepted by the research ethics committee, but data was used only if written consent was obtained at a later time

### Methods

We used electrical velocimetry to estimate cardiac output, stroke volume and heart rate continuously in a noninvasive manner. Electrical velocimetry by ICON® (Osypka Medical, Berlin, Germany and San Diego, CA, USA) uses bioimpedance cardiography technology to detect small changes in electrical current by four electrodes placed on the forehead and on the left side of the neck, thorax and leg [15]. The major contributor to the pulsating alterations in electrical impedance is changes in the flow of the ascending aorta. The rate of change in impedance between systole and diastole is used to calculate an estimate of left ventricular output. Previously, electrical velocimetry has been shown to have similar precision as echocardiography in term and preterm newborn infants, and little bias when compared to echocardiography given that signal quality index ≥ 80% [16,17] and in infants undergoing therapeutic hypothermia [18].

### Study design

We measured cardiac output, stroke volume and heart rate every minute for six hours. Only measurements with signal quality index ≥ 80% were included. For the group of asphyxiated infants, measurements were initiated when it was decided that the infant met the clinical guideline for therapeutic hypothermia or when our neonatal transport team showed up at the local hospital for transfer to the NICU at Rigshospitalet. Data were transferred from the electrical velocimetry device and clinical data as well as data from routine blood samples were collected from patient records after parental consent.

For the control group, monitoring began when the infant was approximately 30 minutes old and continued for six hours. The infant stayed with the parents during the monitoring period and there were no restrictions on how to handle the infant.

### Cardiac output in healthy newborns

In the healthy newborn infants, cardiac output measurements from each infant were collected in 30-minute bins. A mean for each 30-minute bin was calculated and plotted against postnatal age.

### Comparison between asphyxiated and healthy newborns

When the asphyxiated infants reached the targeted temperature (33.5 degrees Celsius), heart rate, stroke volume and cardiac output were fairly stable. Therefore, a mean value for each parameter was calculated for the whole observation period. In five asphyxiated infants, measurements began while the infants were normothermic. Therefore, the measurements from these five infants were divided in a ‘normothermic period’, prior to the active cooling phase, and a ‘hypothermic period’ from 30 minutes after the target temperature was reached. Results from the normothermic period (available in five infants) as well as the hypothermic period (available in all 15 infants) were compared to the control group (10 infants).

### Relation between lactate level and cardiac output

To describe the rate of disappearance of lactate, lactate level was plotted against postnatal age. The relation was best described by a linear relationship with a constant slope till lactate reached a level of 2 mmol/l, where the curve flattened. We excluded all values below 2 mmol/l and performed a linear regression. Rate of disappearance of lactate was estimated as the slope of the regression line and plotted against cardiac output in the first 6 hours of life to determine if the two parameters were related.

### Statistics

The differences between asphyxiated and healthy infants were tested by t-tests. A paired t-test was used to compare the parameters prior to and during hypothermia in the five infants where this was possible. For non-parametric data, the Mann-Whitney U test was used, and Fisher’s exact test was used to compare between dichotomous outcomes. For comparison between rate of disappearance of lactate and cardiac output, Pearson’s correlation was performed. SPSS 22.0 was used for statistical calculations. A p-value below 0.05 was considered significant. No correction for multiple comparisons were used, since conclusions were only drawn as regard one hypothesis.

## Results

### Patients

In the asphyxiated group, four infants were born at Rigshospitalet and 11 infants were born at other hospitals and transported by our team. The antecedents of perinatal asphyxia were cephalo-pelvic disproportion (9), uterine rupture (2), fetal bradycardia of unknown reason (3) and meconium aspiration (1). Five of the infants were delivered by acute caesarian section. During resuscitation 12 infants received fluid boluses, two infants received adrenaline and 11 were intubated (Table 1). Mean postnatal age at initiation of monitoring was 4 hours [1–6,8 hours]. During the monitoring period, six infants had signs of circulatory insufficiency and were treated with dopamine [2–20 microg/kg/min] and one infant received norepinephrine on top of dopamine. We did not observe any difference in cardiac output between infants receiving inotropy and those who were not (128±19 vs. 143±29ml/kg/min, p = 0.281). The lowest mean arterial blood pressure was 44 mmHg [34–60 mmHg], mean hemoglobin was 9.6 mmol/L [5.9–12.4 mmol/L] and three infants received blood. Eight infants had urine output during the monitoring period, however, we would not expect diuresis this shortly after birth. Seven infants were treated with mechanical ventilation during electrical velocimetry monitoring, and five infants were treated with continuous positive airway pressure. The highest Thompson score [19] ranged between 3 and 17, median 8.

**Table 1:** Clinical characteristics.

	Control (n = 10)	Asphyxia (n = 15)	p-value
**Gestational age (weeks)**	38.8±0.8	40.1±1.6	0.031
**Birth weight (g)**	3383±199	3738±588	0.044
**Boys / girls (boys %)**	6/4 (60)	5/10 (33)	0.241^a^
**Apgar 1min (median)**	10 [7–10] (n = 10)	1 [0–4] (n = 14)	<0.001^b^
**Apgar 5 min**	10 [7–10] (n = 10)	3 [0–5] (n = 13)	<0.001^b^
**Apgar 10 min**	-	4 [0–7] (n = 11)	
**Cord-pH**	7.33±0.05 (n = 9)	7.02±0.16 (n = 14)	<0.001
**Cord-BE**	-1.0 ± 2.1 (n = 9)	-11.8±5.8 (n = 12)	<0.001
**Cord-Lactate**	2.1±0.69 (n = 7)	11.4±3.1 (n = 5)	0.002

T-test. Otherwise

^a^Fisher’s exact test and

^b^Mann-Whitney U test.

### Cardiac output in healthy newborns

**Fig 1:**
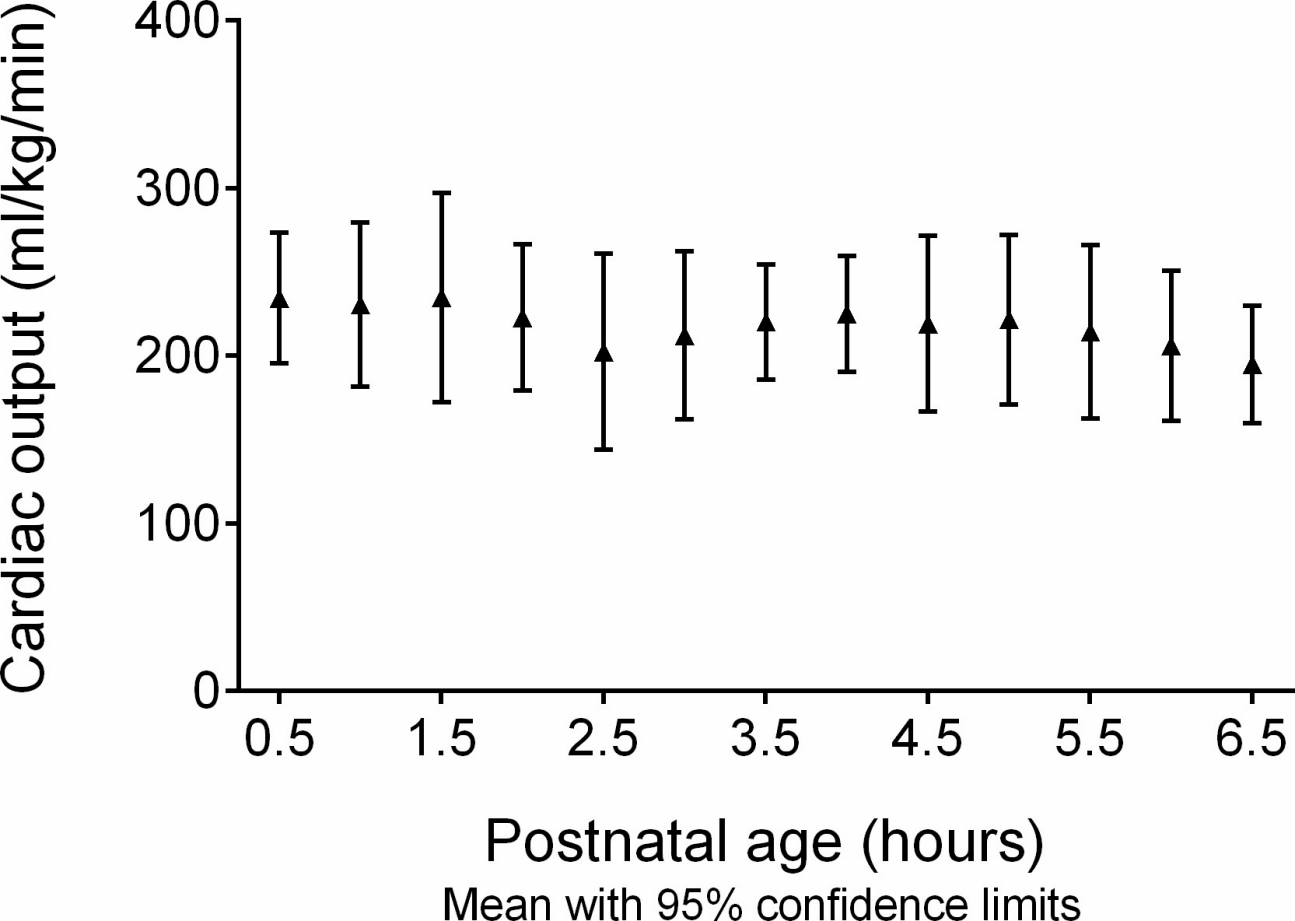
Cardiac output in healthy control infants 0.5 to 6.5-hour postnatal age. Cardiac output was stable in the first 6.5 hours postnatally in 10 healthy control infants delivered by elective caesarian section. Results are given as mean with 95% confidence interval.

In the control group cardiac output was stable in the first 0.5 to 6.5 postnatal hours (Fig 1).

### Comparisons between asphyxiated and healthy infants

**Fig 2:**
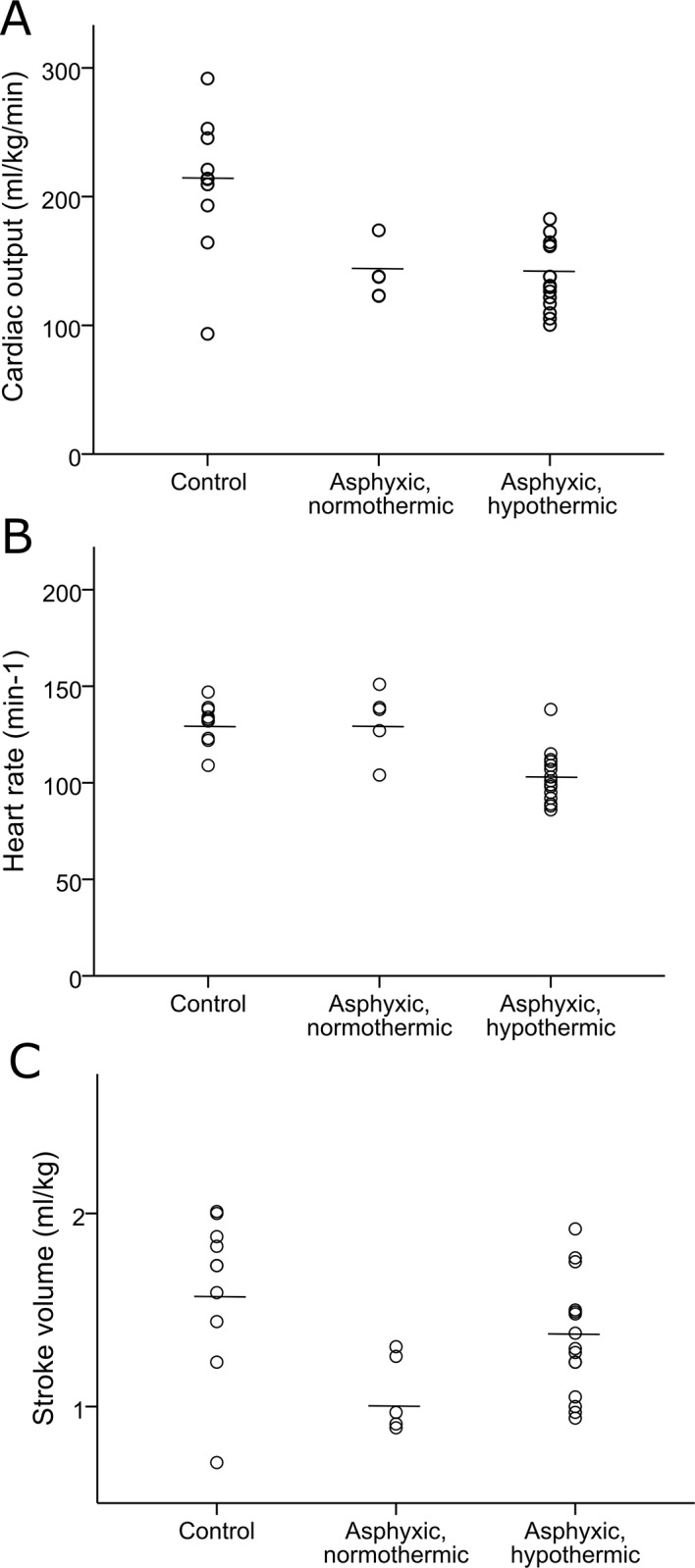
**Comparison of (A) cardiac output, (B) heart rate, and (C) stroke volume between healthy control infants (n = 10) and normothermic asphyxiated infants (n = 5) and hypothermic asphyxiated infants (n = 15).** The lines represent mean values.

In the five asphyxiated infants, where monitoring was initiated during normothermia, both cardiac output and stroke volume were impaired prior to hypothermia, when compared to the control group (cardiac output: 139±21 ml/kg/min vs. 210±54 ml/kg/min, p = 0.015. Stroke volume: 1.07±0.20 ml/kg vs. 1.62±0.40 ml/kg, p = 0.014). Heart rate was comparable in the two groups (132±18 min^-1^ vs. 131±11 min^-1^, p = 0.903) (Fig 2A–2C).

During hypothermic therapy, cardiac output decreased (from 139±21 ml/kg/min to 124±28 ml/kg/min, p = 0.002). This decrease in cardiac output was associated with a trend towards a fall in heart rate (132±18 min^-1^ to 98±11 min^-1^, p = 0.097) whereas stroke volume tended to increase (1.07±0.20 ml/kg to 1.28±0.30 ml/kg, p = 0.140).

Compared to the control group, the hypothermic asphyxiated infants had significantly lower cardiac output (137±26 ml/kg/min vs. 210±54 ml/kg/min, p<0.001) and heart rate 103±13 min^-1^ vs. 131±11 min^-1^, p<0.001) and a tendency to lower stroke volume (1.35±0.30 ml/kg vs. 1.62±0.40 ml/kg, p = 0.075) (Fig 2A–2C).

### Relation between lactate level and cardiac output

**Fig 3:**
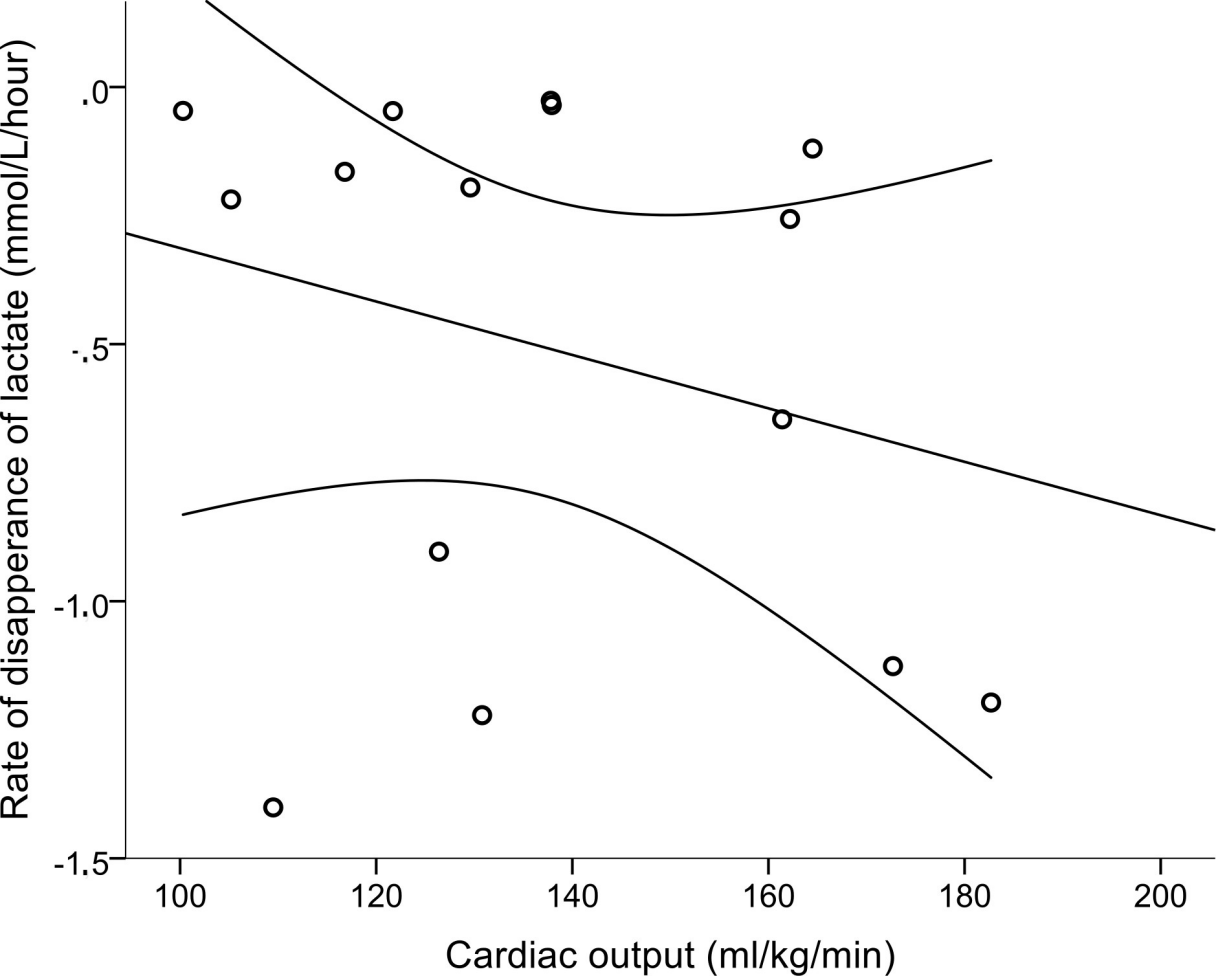
Relation between rate of disappearance of lactate and cardiac output in hypothermic asphyxiated infants. Regression line and 95% confidence limits, R^2^ = 0.067.

In the hypothermic asphyxiated infants, the relationship between rate of disappearance of lactate and cardiac output was positive as hypothesized but far from statistically significant (Fig 3) (regression coefficient -0.259, p = 0.350). Time to reach a lactate level below 2 mmol/l was below 12 hours in six cases, and between 24 to 72 hours in the rest. Rate of disappearance of lactate correlated well with highest Thompson score (r = 0.601, p = 0.018), but not with the highest value of alanine aminotransferase as a parameter of liver affection (r = 0.309, p = 0.329).

## Discussion

The results of this study support that infants with perinatal asphyxia do have lower cardiac output than healthy newborn infants, but the rate of disappearance of lactate was not statistically significantly related to this.

To our knowledge this is the first study that monitored cardiac output continuously prior to and during the first hours of therapeutic hypothermia in asphyxiated newborns. Echocardiography has previously been used to evaluate cardiac function non-invasively in newborns after asphyxia [5–7,9,20]. Compared to echocardiography, electrical velocimetry has the ability to measure changes in cardiac output continuously and the method has recently been used to determine normative data of cardiac output and stroke volume in the first 15 minutes of life in healthy newborns [21].

### Cardiac output in healthy newborns

We demonstrated a cardiac output of approximately 210 ml/kg/min in our control group. This is in line with other studies using both electrical velocimetry [21] and echocardiography [5,20,22,23]. Therefore, we do not think that the fact, that the children in our control group had lower gestational age and birth weight than children in the asphyxiated group, weaken the support for the idea, that asphyxia tend to compromize cardiac output in the hours and perhaps days after birth. The difference in gestational age and birth weight was caused by the fact that we chose a control group of infants delivered by elective caesarean section.

### Cardiac output in asphyxiated infants

Cardiac monitoring was initiated prior to therapeutic hypothermia in five infants, and as expected, we observed a tendency to a decrease in heart rate during initiation of active hypothermia [24].

Prior to active hypothermia, cardiac output was lower in the asphyxiated infants than in the control group. Reduced cardiac output has previously been described in normothermic asphyxiated infants by echocardiography within the first days of life [6,9,10]. Therapeutic hypothermia was not clinical routine while these studies were done and the first measurements were performed at a later time than in our study, however the estimate of cardiac output in our population prior to hypothermia was comparable with the first measurement at 6–12 hours of age in the study by Van Bel et al [6].

When the temperature was lowered, cardiac output decreased due to a fall in heart rate; at the same time stroke volume tended to increase, indicating sufficient diastolic and systolic function. Based on these observations, the lower cardiac output, described in this and in previous studies [6,9,10] may rather reflect a reduced metabolic rate after the hypoxic insult rather than a compromised cardiac function.

Besides the documentation of a fall in heart rate caused by hypothermia [24], neither stroke volume nor cardiac output have been measured continuously during the active cooling phase in infants; but our results match an experiment performed in newborn piglets [25].

Most of the effect of hypothermia on cardiac output is based on observations during the rewarming phase, where it has been shown that heart rate, stroke volume and cardiac output increases [7,18,22,23]. But would we expect to observe the same, but opposite, effect on cardiac output during the active cooling phase? In studies of normothermic asphyxiated infants, cardiac output and stroke volume were most affected in the first days of life and then tended to ‘normalize’ [6,10]. We observed that cardiac output was reduced by 10% during cooling, whereas a study based on the rewarming phase found an increase in cardiac output by 50% [7]. It has been shown that central venous saturation increases while the temperature is lowered in pigs as an indicator of reduced metabolic rate during hypothermia [26]. Therefore, we cannot simply translate the effect of the rewarming phase to that of the active cooling phase, where metabolic rate may be reduced due to the hypoxic insult.

In line with previous studies [5,9,20,22,23], the asphyxiated infants had lower cardiac output and heart rate during hypothermia compared to the healthy newborn infants in our control group.

### Relation between lactate level and cardiac output

A low rate of disappearance of lactate acidosis is associated with higher mortality [11,12] and it has been suggested that raised lactate level may serve as a surrogate marker for suboptimal hemodynamic status of term asphyxiated newborns treated with hypothermia [14]. Lactate levels may reflect reduced perfusion of the organs and we therefore hypothesized that compromised cardiac output in asphyxiated infants would be related to the rate of disappearance of lactate. The support for this hypothesis, however, was very weak. Although the relation was in the expected direction, with a low cardiac output being associated with a low (numeric) rate of disappearance, the relation was weak and in some infants the rate of disappearance was very high in spite of a low cardiac output indicating that this was sufficient to meet the metabolic needs. This is in line with the reasoning above that reduced cardiac output may reflect a reduced metabolic rate rather than a compromised cardiac function.

In fairness, prolonged lactate acidosis may also be explained by microcirculatory dysfunction with regional tissue hypoxia, anaerobic glycolysis seen in response to stress or delayed lactate clearance from the liver [27].

Based on our observations, rate of disappearance of lactate may rather indicate the degree of asphyxia and in support of this, rate of disappearance of lactate correlated well with highest Thompson score.

### Limitations

The study was a single center, observational study with a limited number of infants included. Perinatal asphyxia is always an acute, unprepared and critical situation and it is well described that seeking consent for entry into a trial may aggravate the parents’ distress [28] and the quality of consent in this situation is also low [29]. Therefore, we considered a minimal risk, non-interventional study with deferred consent the best compromise, and decided not to include other measurements in the protocol. This leaves us with limited data that cannot be extrapolated to a general description of the whole group of asphyxiated infants. The study may generate questions regarding the cardiac function in this group of critically ill infants in this field with limited data available.

Our intention was to measure cardiac parameters prior to the active cooling phase. This was, however, only obtained in five out of 15 cases. In most of the cases the temperature was already near the target of 33.5 degree Celsius solely by passive cooling during initial transport, and therefore our results concerning the pre-cooling phase and the effects of cooling should be valued carefully.

The measurements were not blinded to the physicians, this could possible introduce a bias if e.g. use of inotropy was based on the measurements. However, our study was not planned to describe interventions based on the cardiac output measurements and our experience was that the physicians did not include the values in their evaluation of the infants. This is most likely because electrical velocimetry was not implemented as a routine monitoring tool. However, the method is technically simple and circulatory insufficiency is common in asphyxiated newborns, and therefore a future study with focus on the clinical interventions based on cardiac output measurements would be interesting.

Also, grade of hypoxic-ischemic encephalopathy was not blinded to the persons performing data analysis which potentially could introduce bias. However, as prespecified in the protocol, we excluded only electrical velocimetry measurements with signal quality index < 80%.

One question that may be raised is whether the infants had a persistent ductus arteriosus–or maybe even a closure of ductus arteriosus in the measurement period–and whether this might affect our measurement of cardiac output and stroke volume. Since perinatal asphyxia and hypothermia do not seem to affect closure of ductus arteriosus in newborns [7] and piglets [25], we would not expect the occurrence of an open ductus arteriosus to differ among the two groups. Theoretically, electrical velocimetry may be influenced by a hemodynamically significant persistent ductus arteriosus; and some find that a significant patent ductus arteriosus introduces a bias of the measurements [17] whereas others do not [16].

Finally, it would have been interesting to have a measure of the cardiac output throughout the whole cooling period and the rewarming phase as well.

## Conclusion

Our results support the idea that asphyxiated newborn infants have lower cardiac output prior to and during therapeutic hypothermia. An association between disappearance of lactate acidosis and low cardiac output, however, was not confirmed and a low rate of disappearance of lactate may rather be an indicator of organ injury due to asphyxia. The observed lower cardiac output may reflect a reduced metabolic rate in the asphyxiated newborn infants.
